# Does internet addiction affect the level of emotional intelligence among nursing students? A cross-sectional study

**DOI:** 10.1186/s12912-024-02191-6

**Published:** 2024-08-13

**Authors:** Heba Emad El-Gazar, Hanaa Elgohari, Ahmed Loutfy, Mona Shawer, Ahmed Hashem El-Monshed, Mennat Allah G. Abou Zeid, Mohamed Ali Zoromba

**Affiliations:** 1 Nursing Administration Department, Faculty of Nursing, Port-Said University, Port-Said, Egypt; 2 Department of applied statistics, Faculty of commerce, Mansoura University, Mansoura, Egypt; 3 Department of Nursing, College of Health Sciences, University of Fujairah, Fujairah, UAE; 4 Pediatric Nursing Department, Faculty of Nursing, Beni-Suef University, Beni-Suef, Egypt; 5 Nursing Education and Advanced Practice Lead, King’s College Hospital London-Jeddah, Jeddah, Saudi Arabia; 6 Technical Institution of Nursing, Mansoura, Egypt; 7 Department Nursing, College of Health and Sport Sciences, University of bahrain, Manama, Bahrain; 8 Department of Psychiatric and Mental Health Nursing, Faculty of Nursing, Mansoura University, Mansoura, Egypt; 9 College of Nursing, Prince Sattam Bin Abdulaziz University, Al-Kharj, Saudi Arabia; 10 Administration Department, Faculty of Nursing, Ain Shams University, Cairo, Egypt; 11 Faculty of Nursing, Mansoura University, Mansoura, Egypt; 12 Faculty of Business Administration, Horus University, New Damitta, Egypt

**Keywords:** Emotional intelligence, Internet addiction, Nursing students

## Abstract

**Aim:**

To examine the effect of internet addiction on emotional intelligence among nursing students. Internet addiction, especially among nursing students, is an ongoing and urgent issue globally. Despite studies acknowledging its negative effects, the specific impact on the emotional intelligence of college students is not completely explored and needs more investigation.

**Design:**

A correlational cross-sectional descriptive study.

**Methods:**

Participants included 180 nursing students from Mansoura University, Egypt. They completed the Socio-demographic sheet, Internet Addiction Test, and Schutte Self-Report Emotional Intelligence. Descriptive statistics, Pearson correlation, and regression analysis were conducted to analyze the data.

**Results:**

Nursing students experience a moderate level of internet addiction and a neutral level of emotional intelligence. A significant negative relationship was established between internet addiction among students and their emotional intelligence (*r* = − 0.53, *p* < 0.001) and its four facets: perception of emotion, managing own emotions, managing others’ emotions, and utilization of emotion. Regression analysis showed that internet addiction had a negative effect on emotional intelligence among nursing students (β = − 0.553, *p* < 0.001) and explained 30.5% of the variance of emotional intelligence among nursing students.

**Conclusions:**

The study findings suggest that internet addiction negatively impacts the emotional intelligence of nursing students, which is crucial for effective interpersonal relations and professional competence in nursing.

**Implications:**

The study underscores the need for educational programs to raise awareness about the hazards of excessive internet use and to promote activities that mitigate internet addiction.

**Supplementary Information:**

The online version contains supplementary material available at 10.1186/s12912-024-02191-6.

## Introduction

The ubiquity of the internet has led to its integral role in various aspects of daily life, including communication, commerce, education, and leisure, which has become particularly pronounced among nursing students. Their extensive reliance on the internet is believed to be a consequence of their developmental phase, where the formation of identity and meaningful social connections are paramount [1]. This increased dependence is not without its risks; studies suggest a higher propensity for internet addiction (IA) within this demographic, comparable to the addictive behaviors seen in computer gamers [2, 3]. Evidence indicates that approximately 12% of these individuals display addiction patterns akin to substance dependencies, with significant consequences on mental health and social functioning [4]. This phenomenon is prevalent across diverse age groups and social strata. It tends to affect adolescents and young adults most profoundly. This underlines the importance of understanding and addressing the implications of internet addiction in contemporary society [5].

With the pervasive integration of the internet into daily life, the phenomenon of IA among nursing students demands attention due to its potential to impact their academic and future professional performance. IA, characterized by an uncontrollable urge to remain online, leads to detrimental consequences in various aspects of life, including the potential to impair emotional intelligence (EI) [6].

EI, a multifaceted capability, encompasses more than social skills; the synthesis of affective and cognitive processes enables individuals to perceive, understand, and regulate emotions to promote emotional and intellectual growth [7]. Within the nursing profession, EI is paramount as it underpins the empathetic and interpersonal skills essential for patient care and teamwork. Hence, EI’s role extends beyond mere social skills; it is a foundational skill that impacts professional competence and quality of care in nursing practice [8].

The foundation of EI can be traced back to Thorndike’s concept of social intelligence in 1927. However, it only gained substantial recognition with Goleman’s seminal work in 1995 and the defining work of Salovey & Mayer, (1990) [9]. EI, as understood today, involves monitoring one’s own and others’ emotions, discerning between them, and utilizing these insights to guide thoughts and actions, thereby linking emotional and cognitive functions to enhance decision-making. Adolescents with high EI tend to have a richer repertoire of coping strategies, often opting for more adaptive approaches. Mayer and Salovey further refined EI into four distinct dimensions: self-awareness of emotions; recognition of others’ emotions; regulation of emotions; and leveraging emotions to improve cognitive processes and performance [10]. This multifaceted model emphasizes the integral role of emotional processing in overall social intelligence [11–13].

## Background

Since the internet has become a vital component in human lives, particularly nursing students, and an additional factor that affects the lives of individuals’ lives. Therefore, it was necessary to study the extent to which this factor affects individuals’ lives. This aligns with the World Health Organization’s, (2015) discussion on the need to conceptualize and define the scope, phenomenology, and typology of disorders related to excessive use of the internet, smartphones, computers, and similar devices. These disorders share signs and symptoms with substance use disorders and “behavioral addictions” [14].

Nursing students are at a unique crossroads; they are both students, who may be at risk for increased internet usage due to academic pressures and social influences, and budding healthcare professionals for whom the internet is a crucial source of information and a platform for professional development. A study suggests that students with greater academic challenges may be at a higher risk of increased internet usage, which can potentially lead to declines in academic performance [15]. On the other hand, another study showed that the use of social media among nursing students has been noted to enhance learning both formally and informally [16]. While these factors highlight the importance of the internet in their academic and professional lives, further research is needed to establish a direct link between these usage patterns and a higher risk of internet addiction specifically among nursing students.

Excessive online behavior can sometimes serve as an escape from everyday life, leading to a detachment from reality. For some individuals, cyberspace can become a realm where they act out their imaginations and struggles, creating a distinct world separate from their real-life experiences. This detachment can interfere with their ability to engage with their own and others’ emotions, thereby affecting their thinking and actions [17]. Research has shown that problematic internet use can lead to various negative outcomes, including behavioral issues and interference with daily activities [18, 19]. For nursing students, this excessive use of the internet may lead to social withdrawal and other problematic behaviors [15].

Internet users could appreciate features that let them connect with others, communicate, and exchange ideas via chat rooms, social networking sites, or “virtual communities.” All of these online activities have a negative impact on people’s capacity to focus, perceive, and think, as well as their daily activities which leads to an inability to control emotions, negative or positive, throughout the addictive behaviors. Spending a lot of time and money on virtual activities makes people less motivated to engage in social and academic activities and reduces productivity [20].

There are several studies discussed a significant relationship between the use of the internet and psychiatric symptoms like aloneness, obsession, and depression [15, 21, 22]. Hamissi et al., (2013) mentioned that IA affects social activities, and psychological health in previous years [3]. Accordingly, nursing students who used the internet experienced academic difficulties [15]. Studies have highlighted the protective role of EI against IA, suggesting that individuals with higher EI are better equipped to manage their internet use and avoid addictive behaviors. For instance, a study by Fernández-Martínez et al. (2023) and Arbabisarjou et al. (2016) found that higher levels of emotional clarity and emotional repair were associated with lower levels of IA among nursing students [23, 24]. This evidence underscores the importance of developing EI to mitigate the risks associated with IA. Given these points, it is crucial to investigate how IA affects the EI levels of nursing students and the implications for their personal development and professional training. The current study aimed to provide a comprehensive overview of the potential relationship between IA and EI within the nursing student population, thereby informing strategies to enhance EI and reduce IA among these students.

Specific objectives:

- Examine the level of internet addiction among nursing students.
- Determine the level of emotional intelligence among nursing students.
- Investigate the relationship between internet addiction and emotional intelligence among nursing students.
- Assess the impact of internet addiction on the facets of emotional intelligence (perception, managing own and others’ emotions, and utilization).
- Evaluate the influence of demographic factors (age, gender, residence, academic year, GPA) on internet addiction and emotional intelligence.

## Methods

### Study design

A descriptive cross-sectional research design was performed to investigate the effect of internet addiction on emotional intelligence among nursing students. This study was reported according to the STROBE checklist.

### Participants

The sample size was determined using G*Power software version 3.1.9.7 for regression analysis [25]. According to the following parameters: 95% power, an alpha set at 0.05, a medium effect size set at 0.15 [26], and 6 predictors, and a sample size of over 146 students were needed to be approached. An additional 44 students need to be enrolled to account for a predicted dropout rate of 30% [27].

A stratified sampling technique was used to estimate the number of contributing students according to their academic year, with categories ranging from the first to the fifth year. To minimize selection bias, students were selected from each academic year stratum proportionally. Within each stratum, convenience sampling was applied to recruit students. This approach ensured that each academic year was adequately represented while facilitating the practical recruitment of participants. The inclusion criteria were as follows: the study targeted Egyptian university nursing students, aged from 18 to 25 years old, including both males and females from the second to the last education year of their educational program. Non-Egyptian students and those who refused to complete the questionnaires were excluded. Of the 191 forms distributed, 184 were returned, and 4 were invalid. As a result, the study’s final sample size was 180, and its response rate was 94.2%.

### Measurements

#### Demographic characteristics form

Demographic characteristics included gender, age, residence, academic level, and Grade Point Average (GPA), which was categorized as follows: acceptable (2:2.2), good (2.3:2.9), very good (3:3.5), and excellent (3.6: 4) according to students’ affairs policy.

#### Internet addiction

The Arabic version of the Internet Addiction Scale was used to measure the severity of self-reported compulsive use of the internet among university students [28]. This scale was originally developed by Young (1998) and consists of 20 items on a 5-point Likert scale from zero (never) to four (always). Cumulative scores ranged from 20:100, with the higher the scores, the greater the dependence on the internet [29].

#### Emotional Intelligence

Schutte Self-Report Emotional Intelligence Test (SSRIET); Schutte et al., (1998) was used to measure levels of emotional intelligence among students [30]. The SSRIET comprises 33 self-report statements covering four facets of emotional intelligence: perception of emotion (10 items), managing own emotion (10 items), managing other emotions (7 items), and utilization of emotion (6 items) [12]. Participants responded on a Likert scale Likert scale from strongly disagree (1) to strongly agree (5). High scores on all items collectively direct high levels of EI.

Researchers translated The SSRIET into the Arabic language following the translation-back-translation technique [31]. Initially, the SSRIET scale was translated from English to Arabic by the principal author, and subsequently, a back-translation from Arabic to English was conducted by a proficient translator. This new version was meticulously compared to the original English scale, and necessary corrections were implemented to ensure close alignment between the translated version and the original English scale. Following this, a panel of five nursing professors reviewed the translation to ensure terminological equivalence and clarity. Some words were adjusted to fit the Egyptian context. The translated scales were then pre-tested for validity. Confirmatory factor analysis (CFA) provided evidence for the construct validity of the study scales and factor loadings and measurement indicators confirmed the validity and reliability of the measurement instruments used in this study (Appendix).

The internal consistency of the Internet Addiction Scale, adapted to Arabic, was tested and verified using Cronbach’s α. The Cronbach’s α for the Internet Addiction Scale was 0.95, and for the SSRIET, it was 0.96. Both values exceed the suggested threshold of 0.7, indicating the high reliability of the scales used in this study [32].

### Data collection

Data were collected between March and May 2023. Researchers recruited participants by visiting classrooms, explaining the study’s purpose, the voluntary nature of participation, and ensuring anonymity. The self-administered questionnaires, which included a cover letter detailing this information, were then distributed to the students. Participants were given sufficient time to complete the questionnaires, and the completed questionnaires were collected by the researchers on-site. This approach ensured that all participants were fully informed and that the data collection was conducted efficiently and ethically.

### Statistical analysis

Statistical analysis was accomplished using IBM SPSS 28 and AMOS version 24. Frequencies and percentages were used to describe the categorical demographic characteristics of the participating students, such as gender, age, residence, academic year, and GPA. Means and standard deviations were calculated for continuous numerical variables. Confirmatory Factor Analysis (CFA) was applied to assess the construct validity of the study scales. Additionally, to strengthen the measurement model, both discriminant and convergent validities were evaluated. To find differences in the study variables based on demographic traits, a one-way analysis of variance (ANOVA) was used to determine the differences between educational levels and GPA, or other variables with more than two groups. Independent sample t-tests were used to compare differences between two groups, such as gender and residence. The Pearson (r) correlation coefficient was used to determine the relationships between internet addiction and emotional intelligence. Linear regression analysis was used to test the effect of internet addiction on EI among nursing students. Furthermore, we have conducted additional diagnostics to ensure the robustness of our analysis. The Durbin-Watson statistic (1.561) suggests no significant autocorrelation in the residuals, indicating that the assumption of independence of observations was met. Additionally, the tolerance and variance inflation factor (VIF) values (both equal to 1) indicate no issues with multicollinearity among the predictor variables.

### Common method bias (CMB)

The data in this study were self-reported and collected from a single source, which increased the threat of CMB. To alleviate the threat of CMB, the suggestion of Podsakoff et al. (2012) was followed; scales-items were randomly interspersed into the questionnaire, informing the respondents of study design and purpose, adherence to anonymity, confidentiality and purely voluntary participation, and pre-tested the study scale to ensure the clarity of scale items [33]. In addition, Harman’s single-factor test was used to statically assess the presence of CMB [34], which displayed that a solitary factor verified 38.4% of the variance, below the cut–off point of 50% [35]. This showed that CMV was not a major issue in this study.

### Ethical considerations

This study was approved by the Nursing Ethics Committee, Faculty of Nursing, Mansoura University (Ref. p.0429). All participants signed the informed consent form and voluntarily completed the questionnaires without any incentive. In addition, all the participants’ information remained strictly confidential and anonymous.

## Results

Slightly more than half of the participants were female (51.1%), aged more than 20 years (51.1%), and from urban (57.8%). Nearly a quarter (23.9%) of participants were fourth-year students and 39.4 scored a very good GPA. There is no difference in studied variables according to the sociodemographic data of studied students (Table 1).

**Table 1:** Participants’ demographics and differences in study variables (*n* = 180)

Characteristic	Category	Frequencies		Internet Addiction		Emotional Intelligence	
		No	%	Mean (SD)	t/F (*p-value*)	Mean (SD)	t/F (*p-value*)
Age (years)	*≤* 20	88	48.9	2.59 (0.59)	t = 1.89 (0.06)	3.19 (0.68)	t= -1.13 (0.26)
	> 20	92	51.1	2.44 (0.50)		3.31 (0.63)	
Gender	Men	88	48.9	2.52 (0.55)	t = 0.244 (0.81)	3.25 (0.62)	t= -0.12 (0.91)
	Women	92	51.1	2.50 (0.55)		3.26 (0.69)	
Residence	Urban	104	57.8	2.48 (0.50)	t= -1.09 (0.28)	3.29 (10.1)	t = 0.74 (0.46)
	Rural	76	42.2	2.57 (61.8)		3.21 (0.73)	
Academic Year	First	37	20.6	2.54 (0.60)	F = 1.69 (0.15)	3.14 (0.70)	F = 0.56 (0.69)
	Second	32	17.8	2.63 (0.58)		3.28 (0.63)	
	Third	36	20.0	2.63 (0.54)		3.21 (0.59)	
	Fourth	43	23.9	2.40 (0.48)		3.33 (0.66)	
	Fifth	32	17.8	2.38 (0.53)		3.32 (0.71)	
Grade Point Average	Acceptable	14	7.8	3.05 (0.57)	F = 0.077 (0.105)	2.25 (0.33)	F = 2.322 (0.077)
	Good	58	32.2	3.13 (0.63)		2.62 (0.58)	
	Very good	71	39.4	3.31 (0.69)		2.53 (0.55)	
	Excellent	37	20.6	3.44 (0.63)		2.43 (0.55)	

t, Independent Sample t-test; F, ANOVA test; SD, Standard Deviation; *p*-value, significance level

As shown in Table 2, the mean score of participants’ internet addiction was 2.51 ± 0.55 out of 4, indicating a moderate level of internet addiction among nursing students. The mean score of participants’ emotional intelligence was 3.26 ± 0.66 out of 5, indicating a neutral level of emotional intelligence among studied students. The mean score for “managing own emotions” was the lowest among the four facets of emotional intelligence (2.83 ± 0.74). The Pearson correlation results showed a moderate negative relationship between IA and EI and this relationship was statistically significant (*r* = − 0.53, *p* > 0.001) and its four facets; perception of emotion, managing own emotions, managing others’ emotions, and utilization of emotion (*r* = -0.48, -0.46, -0.52, -0.53, *p* > 0.001; respectively).

**Table 2:** Cronbach’s α, Means, Standard deviations, and correlations (*N* = 180)

Variable	α	Mean ± SD	1	2	3	4	5
1. IAT Total Score	0.95	2.51 ± 0.55	1				
2. EI Total Score	0.96	3.26 ± 0.66	− 0.53**	1			
3. Perception of Emotion	0.87	3.43 ± 0.54	− 0.48**	0.86**	1		
4. Managing own Emotions	0.91	2.83 ± 0.74	− 0.46**	0.95**	0.78**	1	
5. Managing other Emotions	0.86	3.45 ± 0.77	− 0.52**	0.96**	0.72**	0.88**	1
6. Utilization of Emotion	0.80	3.31 ± 0.73	− 0.53**	0.98**	0.80**	0.91**	0.97**

As shown in Table 3, the linear regression analysis revealed a significant negative effect of internet addiction on emotional intelligence among nursing students. The standardized regression coefficient (β) of -0.553 indicates that for every one-unit increase in internet addiction score, there is a corresponding decrease of approximately 0.553 units in emotional intelligence score. This relationship is statistically significant (*p* < 0.001), with a confidence interval (CI) of -1.263 to -0.802, indicating the precision of our estimate. The model indicated that approximately 30.5% of the variance in emotional intelligence scores can be explained by internet addiction. The F-statistic is highly significant (F = 78.269, *p* < 0.001), further supporting the model’s validity.

**Table 3:** The results of linear regression analysis predict the emotional intelligence of the studied students (*N* = 180)

Predicator	Emotional intelligence					
	B	SE (B)	β	t	*P* value	95% CI (lower / Upper)
Constant	156.496	6.004		26.067	< 0.001	144.649 / 168.343
Internet addiction	-1.032	0.117	-0.553	-8.847	< 0.001	-1.263 / -0.802

*R* = 0.553; R^2^ = 0.305; Δ*R*^2^ = 0.302, F = 78.269; *P* < 0.001, SE, standard error; β = standardized regression coefficient; CI, confidence interval

## Discussion

This study intended to examine the effect of IA on EI among nursing students. The results indicated a moderate level of IA among a sample of nursing students. This result is consistent with those reported among Palestinian [36], Nigerian [37], and Iranian university students [38]. Meanwhile, it was higher than those reported among Mexican, Spainish [39], and Japanese university students [40]. This disparity is possibly related to cultural and socioeconomic differences, availability of leisure-time sports activities, and differences in the characteristics of studied students.

The current study found a significant negative relationship between IA and emotional EI among nursing students. The linear regression analysis revealed that for every one-unit increase in the internet addiction score, there is a corresponding decrease of approximately 0.553 units in the emotional intelligence score. This relationship is statistically significant, with internet addiction explaining 30.5% of the variance in emotional intelligence scores. These findings underscore the detrimental impact of IA on EI in this population. This is consistent with previous studies that have highlighted the negative effects of excessive internet use on emotional regulation and interpersonal skills [19, 22, 41]. These results can be explained by Cho & Lee (2017) who concluded that all addictive tendencies interfere with daily life activity, increase loneliness and isolation, and cause personality distortion [42]. Supporting this result, a previous study among secondary schools found that problematic internet use is negatively related to EI [43].

Our results show that all four facets of EI—perception of emotion, managing own emotions, managing others’ emotions, and utilization of emotion—are negatively impacted by IA. This comprehensive impact highlights the need for targeted interventions to help nursing students manage their internet use and develop their emotional skills. Other studies go further and assert that IA causes self-harm behaviors such as depression and suicidal ideation [11, 40, 44]. Compared to other studies, the level of emotional intelligence among our sample of nursing students was neutral, which aligns with findings from studies in Pakistan [45] and the United States [46] but differs from those in Spain [47] and India [48]. This suggests that cultural and educational contexts may influence emotional intelligence, and these factors should be considered when developing interventions.

The moderate negative relationship observed in our study suggests that as nursing students become more addicted to the internet, their ability to perceive, manage, and utilize emotions effectively diminishes. This can have significant implications for their professional competence and the quality of care they provide. This important result calls for urgent actions to decrease the level of IA among nursing students. In addition, universities should prepare educational programs to increase awareness about the hazards of internet addiction. Parents and nursing university administrators should introduce alternatives to the internet such as book readings and leisure-time sports activities.

The study results showed that IA experienced by nursing students did not vary significantly by age, gender, residence, academic year, and GPA. These results are in line with a meta-analysis study that confirmed that the severity of IA did not differ according to students’ sociodemographic variables [49]. However, these results are partially supported by a previous study that showed no difference in IA among nursing students’ sociodemographics except for gender [13, 36]. The results showed no significant between-group differences in age, gender, residence, academic year, and GPA regarding emotional intelligence. The literature showed mixed results regarding personal characteristics and differences in emotional intelligence. A study by Yudes et al. (2022) showed no differences in EI according to gender [43]. However, another study contrasted the study findings and showed that female students had higher EI than male students [50]. Additionally, IA can lead to psychological distress, decrease academic competence, and hinder educational progress, and long-term career goals [51].

Cultural and societal factors play a crucial role in shaping the EI of nursing students. Egypt has a rich cultural heritage with a strong emphasis on family values and collectivism. These cultural traits may play a role in shaping the EI of nursing students, as individuals from collectivist cultures often place high importance on interpersonal relationships, empathy, and social harmony, which are key components of EI. Additionally, societal norms in Egypt may influence internet addiction patterns, as there is a growing trend toward increased internet usage, especially among youth, for social interaction, entertainment, and educational purposes. Therefore, it is crucial to consider these cultural and societal factors when interpreting the results of this study and their potential implications for nursing student populations in Egypt.

The findings of this study have several important implications for nursing education and practice. First, the significant negative relationship between internet addiction and emotional intelligence highlights the need for educational programs to raise awareness about the risks of excessive internet use among nursing students. Developing interventions that promote healthy internet use and enhance emotional intelligence is crucial. Universities should implement strategies to help students balance their online activities with face-to-face interactions and other productive activities. Introducing alternatives to excessive internet use, such as book readings and leisure-time sports activities, can help mitigate the negative impact of internet addiction. Enhancing emotional intelligence through targeted training can improve nursing students’ professional competence and the quality of care they provide.

### Limitations and future research directions

Although this research contributes to the literature on internet addiction and emotional intelligence, some limitations must be considered. First, the cross-sectional design of the study limits the ability to establish causal relationships between the studied variables. While it allows for the identification of associations, it does not provide evidence of causality. To better understand the direction and causality of the relationships observed, further longitudinal and experimental research designs are suggested. Secondly, while the study focused on Egyptian nursing students, the findings may not be generalizable to nursing students in other countries due to cultural and societal differences. The choice of the sample was influenced by practical considerations, such as proximity and ease of data collection. To enhance the generalizability of the findings, future research should include multicentered studies involving nursing students from diverse geographical locations.

## Conclusions

In this study, nursing students experienced a moderate level of internet addiction and a neutral level of emotional intelligence. Additionally, internet addiction and emotional intelligence were not influenced by nursing student characteristics. The Pearson correlation results showed a moderate negative relationship between internet addiction and emotional intelligence, and this relationship was statistically significant.

### Electronic supplementary material

Below is the link to the electronic supplementary material.

Supplementary Material 1

## Data Availability

Upon request for scientific purposes, the researcher of correspondence will provide the dat of the research.
