# Supplementary material for: Does internet addiction affect the level of emotional intelligence among nursing students? A cross-sectional study
Source: BMC Nurs. 2024 Aug 13;23:555. doi: 10.1186/s12912-024-02191-6 (PMC11321225; doi:10.1186/s12912-024-02191-6)
Supplement: Supplementary file 1 — Supplementary Material 1 [file 12912_2024_2191_MOESM1_ESM.docx]

Validity, reliability, and rigor

First, the sample adequacy was assessed through the Kaiser–Meyer–Olkin (KMO) test and Bartlett test of sphericity. The KMO value needs to be > 0.60, and the Bartlett test of sphericity should be significant at *p* < .05. The results showed that the KMO and Bartlett test of sphericity values were 0.917 (*p* < .001) for the internet addiction scale and 0.912 (p < .001) for the SSRIET. Confirmatory factor analysis (CFA) was used to check the construct validity of the measures used. The CFA for the internet addiction scale was χ2 = 735.71, df = 162, χ2/df = 4.54, CFI = 0.91, TLI = 0.91, RMSEA = 0.073 and for SSRIET was χ2 = 2116.21, df = 491, χ2/df = 4.31, CFI = 0.91, TLI = 0.90, RMSEA = 0.075. Furthermore, all of the constructs used in this investigation had factor loadings that exceeded the suggested value of .5 (Hair et al., 2019), providing evidence for the construct validity of the study scales.

As presented in table 4, the factor loadings for the internet addiction scale ranged from .55 to .93, indicating a strong contribution of each item to the overall construct. for the emotional intelligence scale, the factor loadings for its four facets (perception of emotion, managing own emotions, managing others’ emotions, and utilization of emotion) ranged from .51 to .88, demonstrating that each item adequately represents its respective factor. the use of these factor loadings confirms the validity and reliability of the measurement instruments used in this study.

Table 4. Factor Loadings and Measurement Indicators

| Variable | Items | Factors Loading |
| --- | --- | --- |
| Internet addiction scale | Items 1-20 | .55–.93 |
| Emotional intelligence scale | Items 1-33 | .51–.88 |
| Perception of Emotion | Items 1-10 | .52–.80 |
| Managing own Emotions | Items 11-20 | .53–.88 |
| Managing other Emotion | Items 21-27 | .51–.85 |
| Utilization of Emotion | Items 28-33 | .55–.73 |
